# Acquired lymphangiectasia of the lip in a patient with Crohn’s disease^☆^

**DOI:** 10.1016/j.abd.2022.05.010

**Published:** 2023-09-23

**Authors:** Ana Llull-Ramos, Juan Garcías-Ladaria, Inés Gracia-Darder, Aniza Giacaman

**Affiliations:** Department of Dermatology, Son Espases University Hospital, Palma de Mallorca, Spain

Dear Editor,

Acquired lymphangiectasia is a rare complication of lymphatic obstruction. It has been described in association with malignancy and granulomatous diseases, namely orofacial granulomatosis, Crohn’s Disease (CD), and tuberculosis. It must be distinguished from a congenital malformation, generally referred to as lymphangioma.^1^ Herein we describe a case of acquired lymphangiectasia of the lip in a girl with CD, to our knowledge the first case of this association in pediatric age.

An 11-year-old girl was referred to our Dermatology Department due to persistent cheilitis and gingivitis since she was 4 years old. A therapeutic attempt with gingivoplasty had been performed at the age of 8 without success. No histology was available from the intervention. Later, at the age of 9, she had been diagnosed with Crohn’s disease after intestinal biopsy and was treated with azathioprine and infliximab. However, the cheilitis persisted.

Physical examination showed crusty lesions and flaccid blisters on both lips, as well as erythema with fissures on the gums and jugal mucosa (Fig. 1 A and B). A biopsy of the labial mucosa was performed.

**Figure 1:**
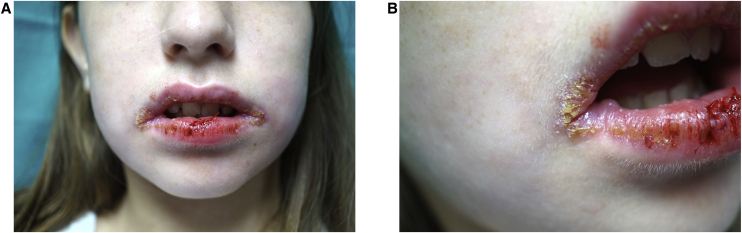
Clinical appearance of the girl’s lips (A and B).

Histology showed epidermal hyperplasia without atypia with lichenoid pattern inflammation and lymphangiectasia (Fig. 2A). The presence of numerous podoplanin-positive subepithelial dilated vessels with a lymphatic appearance is noteworthy (Fig. 2B). Further investigations, including patch testing with the European standard and cosmetics series, and a facial MRI were normal. Topical corticosteroid treatment was prescribed with the intention of reducing the associated inflammation, followed by topical rapamycin 0.2% to decrease the lymphatic component, with no clear improvement after two months.

**Figure 2:**
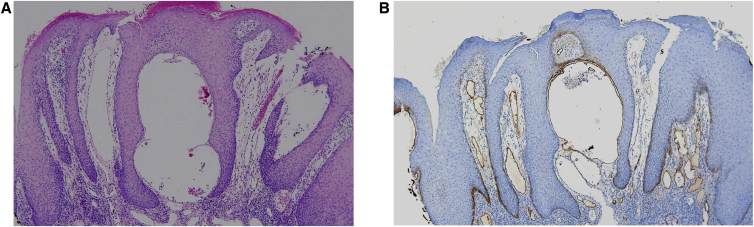
(A) Labial mucosa fragment lined by squamous epithelium with marked acanthosis, spongiosis, hyperkeratosis and no keratinocyte atypia, with a dense lymphoplasmacytic infiltrate in the deeper areas without granulomas, with focal lichenoid change (Hematoxylin & eosin, ×100). (B) Positive immunostaining for podoplanin in subepithelial lymphatic vessels (Hematoxylin & eosin, ×100).

Granulomatous cheilitis presents as orofacial inflammation secondary to non-necrotizing granulomas. It can present isolated or in the context of granulomatous systemic disease. In children, granulomatous cheilitis is more often associated with CD than in adults, being the first manifestation in 5%‒10% of cases of inflammatory bowel disease.^2^, ^3^

Acquired lymphangiectasia in CD is thought to develop as a consequence of chronic granulomatous inflammation. It is described most often in the genital area. In a literature review, we found four cases of acquired orofacial lymphangiectasia associated with CD (Table 1).^1^, ^2^, ^4^ As in the case presented here, in two of these patients no granulomas were observed on histology, and in another case, they were only observed in the second biopsy. The medical treatment could have reduced the inflammation, leaving only the residual lymphangiectasia.^1^, ^2^, ^4^

**Table 1:** Summary of cases of acquired lymphangiectasia of the lip associated with Crohn's disease.

N (Ref)	Year	Age/Sex	LIP histological findings	Treatment	Of outcome
1^1^	2017	18/F	Widely dilated superficial lymphatic channels	Azathioprine, mesalazine, infliximab, 0.03% topical tacrolimus and lip cryotherapy	Good cosmetic result
2^1^	2017	33/M	Superficial dilated lymphatics and patchy inflammation	Triamcinolone injections, 1% topical hydrocortisone cream, oral mesalazine	No treatment needed
3^2^	2020	29/F	1st biopsy: superficial dilated vessels and dense inflammatory infiltrate with a tiny granuloma	Infliximab	Improvement
			2nd biopsy: superficial lymphangiectasia, inflammatory infiltrate and small granuloma		
4^4^	2021	30/F	1st biopsy: proliferation of ectatic lymphatic channels	Methotrexate and infliximab	Improvement
			2nd biopsy: granulomata adjacent to dilated lymphatic channels		
5 (present case)	2021	11/F	Lichenoid pattern inflammation and lymphangiectasia	Azathioprine, infliximab, topical corticosteroids and 0.2% topical rapamycin	No clear improvement

In the absence of granulomatous inflammation, lymphangiectasia can be treated with cryotherapy, sclerotherapy, surgical excision, laser ablation, or photocoagulation.^1^ In our case, topical rapamycin was attempted with no results at 2 months. No cases of acquired lymphangiectasia have been successfully treated with topical rapamycin, but 0.1% topical sirolimus has been effective in cutaneous microcystic lymphatic malformations in children and adults.^5^

In conclusion, the presence of orofacial acquired lymphangiectasia could be an early marker of inflammatory bowel disease even in the absence of granulomatous cheilitis or digestive symptoms. Its diagnosis could help in the early detection of CD.

## Financial support

None declared.

## Authors' contributions

Ana Llull-Ramos: Concepcion; design; acquisition of data; drafting the article; analysis of data; final approval of the version to be published.

Juan Garcías-Ladaria: Acquisition of data; analysis of data; final approval of the version to be published.

Inés Gracia-Darder: Acquisition of data; analysis of data; final approval of the version to be published.

Aniza Giacaman: Acquisition of data; analysis of data; final approval of the version to be published.

## Conflicts of interest

None declared.
